# An asymptotically correct implicit–explicit time integration scheme for finite volume radiation-hydrodynamics

**DOI:** 10.1093/mnras/stae1244

**Published:** 2024-05-11

**Authors:** Chong-Chong He, Benjamin D Wibking, Mark R Krumholz

**Affiliations:** Research School of Astronomy and Astrophysics, Australian National University, Canberra, ACT 2611, Australia; Department of Physics and Astronomy, Michigan State University, East Lansing, MI 48824, USA; Research School of Astronomy and Astrophysics, Australian National University, Canberra, ACT 2611, Australia; ARC Centre of Excellence for Astronomy in Three Dimensions (ASTRO-3D), Canberra, ACT 2611, Australia

**Keywords:** diffusion, hydrodynamics, radiation: dynamics, methods: numerical

## Abstract

Numerical radiation-hydrodynamics (RHD) for non-relativistic flows is a challenging problem because it encompasses processes acting over a very broad range of time-scales, and where the relative importance of these processes often varies by orders of magnitude across the computational domain. Here, we present a new implicit–explicit method for numerical RHD that has a number of desirable properties that have not previously been combined in a single method. Our scheme is based on moments and allows machine-precision conservation of energy and momentum, making it highly suitable for adaptive mesh refinement applications; it requires no more communication than hydrodynamics and includes no non-local iterative steps, making it highly suitable for massively parallel and Graphics Processing Unit (GPU)-based systems where communication is a bottleneck; and we show that it is asymptotically accurate in the streaming, static diffusion, and dynamic diffusion limits, including in the so-called asymptotic diffusion regime where the computational grid does not resolve the photon mean-free path. We implement our method in the GPU-accelerated RHD code quokka and show that it passes a wide range of numerical tests.

## 1 INTRODUCTION

Radiation-hydrodynamics (RHD) plays a significant role in astrophysics, influencing the evolution and energy distribution in various astrophysical systems or phenomena – stellar atmospheres (e.g. Mihalas 1978), planetary atmospheres (e.g. Zhang 2020), core-collapse supernovae (e.g. Skinner, Burrows & Dolence 2016; Radice et al. 2018), star formation in a variety of environments (e.g. Thompson, Quataert & Murray 2005; Krumholz et al. 2009; Rosen et al. 2016; He, Ricotti & Geen 2019; Menon, Federrath & Krumholz 2023), active galactic nuclei and jets (e.g. Davis & Tchekhovskoy 2020), and galactic outflows (e.g. Naab & Ostriker 2017; Zhang 2020). The dynamics of RHD systems vary substantially in a range of scales and physical conditions, parametrized by the typical optical depth and the sound speed of radiation-interacting gas, which determines how radiation is transported. While several numerical techniques exist to solve the RHD equations in various limits, developing methods that are accurate across all regimes and that run efficiently on modern, GPU-based architectures remains an ongoing challenge.

There are well-known difficulties associated with solving the RHD equations numerically for non-relativistic systems. One significant challenge arises from the huge difference in time-scales associated with radiation and hydrodynamics – characterized by the speed of light *c* and gas flow speed *v*, respectively. A second is that realistic RHD problems often contain a huge range of opacities, such that the photon mean-free path may be comparable to the size of the entire simulation domain in some regions, while in others it may be so small as to be impossible to resolve at reasonable computational cost. The RHD equations are well-suited to hydrodynamics-like explicit solution methods in some regimes, but often the source terms coupling the gas and radiation are so stiff that an implicit method must be used to ensure stability.

To deal with these challenges, it is natural to solve both the transport and source terms in the radiative transfer (RT) equations implicitly so that the time-step is not limited by the speed of light. This is the approach adopted by many RHD codes (e.g. Krumholz et al. 2007; Zhang et al. 2011; Jiang, Stone & Davis 2012; Menon et al. 2022). However, this approach suffers from the need for an implicit treatment of the transport term, which is non-local. In multiple dimensions, implicit update of this term is usually accomplished by solving a sparse matrix system, and sparse matrix solvers show limited scalability and performance on modern massively parallel and GPU-accelerated architectures, where the high and unpredictable communication load they involve becomes a performance bottleneck.

Several authors have explored alternative approaches in which the transport and source terms are operator-split, with the former treated explicitly and the latter implicitly (e.g. Jiang, Stone & Davis 2014; Rosdahl & Teyssier 2015; Skinner et al. 2019; Wibking & Krumholz 2022). These schemes generally achieve better scaling and speed, particularly when running in parallel on large numbers of GPUs. One of the disadvantages of this approach is that the explicit treatment of the transport term requires a time-step significantly smaller than the hydrodynamics one (since *c* ≫ *v*), but it is possible to alleviate this problem by subcycling the radiation transport step relative to hydrodynamics. Since the transport equations for radiation are much simpler than those for hydrodynamics, it is possible to carry out many radiation transport updates per hydrodynamic update at a comparable cost. If necessary the cost can be mitigated further using the reduced speed of light approximation (RSLA; Gnedin & Abel 2001; Skinner & Ostriker 2013).

A second challenge in the operator-split approach has received less attention in the literature, but is perhaps even more serious: the need to properly balance the transport and source terms across all asymptotic regimes. This balance is critical because the RT equation behaves as an advection equation in optically thin regimes, but transitions to a diffusion equation in optically thick regimes, and near-perfect cancellations between parts of the source and transport terms are responsible for this behaviour. Efforts to recover this property of the RHD system have thus far mostly involved ad hoc corrections to the Riemann solver or to the source terms to recover the correct asymptotic limit. For instance, Rosdahl & Teyssier (2015) add a ‘trapped photons’ term to the source term to account for diffusion. Skinner et al. (2019) apply corrections to the wave speed in the Riemann solver, scaling down the characteristic speed of the radiation modes in optically thick regimes by a factor of $1/\sqrt{N \tau _{\rm cell}}$, where τ_cell_ is the optical depth per cell, and *N*, chosen empirically, is a small integer. A similar approach is used by Wibking & Krumholz (2022), hereafter [Bibr bib37][Bibr bib37]. Despite these efforts, such corrections are often only partly successful, and their accuracy across a wide range of parameter space has not been tested. Perhaps the most successful (in terms of accuracy) operator-split method published to date is the discontinuous Galerkin implicit–explicit (DG-IMEX) scheme proposed by McClarren et al. (2008), but as we discuss below even this scheme is not accurate in all RHD limits, and it has a number of other undesirable properties as well.

This situation motivates our goal of designing a method that achieves the best of both worlds: accuracy in all RHD regimes that is comparable to that achieved by fully implicit methods, but without the need for poorly scaling communication-intensive operations like sparse matrix solves. In this paper, we describe a method that achieves this goal using a novel time-integration scheme that recovers the proper asymptotic limit in the radiation diffusion regime without requiring non-local implicit updates. Our method is based on a convex-invariant, asymptotic-preserving IMEX approach that is second-order accurate in streaming limit, wherein the transport terms are handled explicitly, while the matter-radiation interacting part is treated implicitly and *locally*, eliminating the need for non-local implicit terms in iteration.

We begin in Section 2 by introducing the full set of two-moment RHD equations to be solved and deriving characteristic numbers and limiting behaviours for them, laying the foundation for our analysis. Then, in Section 3, we present the IMEX scheme. In Section 4, we derive some properties of our scheme and compare to alternative approaches. Finally, we present in Section 5 tests of our implementation, demonstrating its effectiveness and applicability.

## 2 THE RHD EQUATIONS AND NUMERICAL METHODS FOR SOLVING THEM

We begin our analysis by describing the RHD system of equations in Section 2.1, non-dimensionalizing it to obtain characteristic numbers and limiting regimes in Section 2.2, and then using those to gain insight into the challenges of designing numerical methods for RHD and to motivate our approach in Section 2.3.

### 2.1 The RHD system

Our method begins from the fundamental equations of RHD^1^ expressed in an inertial (lab) reference frame. These are


(1) \begin{eqnarray*}
\frac{\partial \boldsymbol{\sf U}}{\partial t}+\nabla \cdot \boldsymbol{\sf F}_{\boldsymbol{U}}=\boldsymbol{\sf S}_{\boldsymbol{U}},
\end{eqnarray*}

where


(2) \begin{eqnarray*}
\boldsymbol{\sf U}=\begin{bmatrix}\rho \\
\rho \boldsymbol{v} \\
E_{\rm gas} \\
E \\
\frac{1}{c^2} \boldsymbol{F} \end{bmatrix}, \,\, \boldsymbol{\sf F}_{\boldsymbol{U}} = \begin{bmatrix}\rho \boldsymbol{v} \\
\rho \boldsymbol{v} \otimes \boldsymbol{v}+p \\
(E_{\rm gas} + p) \boldsymbol{v} \\
\boldsymbol{F} \\
\mathsf {\mathit{ P}}\end{bmatrix}, \,\, \boldsymbol{\sf S}_{\boldsymbol{U}}=\begin{bmatrix}0 \\
\boldsymbol{G} \\
c G_0 \\
-c G_0 \\
-\boldsymbol{G} \end{bmatrix}.
\end{eqnarray*}

are vectors of the conserved quantities, the advection terms, and the source terms, respectively; in the equations above, ρ is the matter density, $\boldsymbol{v}$ is the matter velocity, *p* is the matter pressure, *E*_gas_ is the gas total energy density, *E* is the radiation energy density, $\boldsymbol{F}$ is the radiation flux, $\mathsf {\mathit{ P}}$ is the radiation pressure tensor, and $(cG_0, \boldsymbol{G})$ is the radiation four-force. A significant advantage of working with lab-frame rather than comoving-frame radiation quantities is that these equations are manifestly conservative, a feature that the algorithm we describe below will preserve. As usual in the moment formulation, however, one must adopt a closure relation for the radiation pressure tensor $\mathsf {\mathrm{ \mathit{ P}}}$. There are a wide range of possible closures, and since our scheme is independent of this choice, we will not discuss closure relations further here.

Our next step is to write out the radiation four-force in the mixed-frame formulation, whereby we write the matter–radiation exchange coefficients in the comoving frame, where they are simplest, while all other quantities remain in the lab frame. We assume that the emitting matter is in local thermodynamic equilibrium, so its emissivity is proportional to the Planck function, and we neglect scattering. To order *v*^2^/*c*^2^ the result, expressed in index notation and adopting the Einstein summation convention, is (Krumholz et al. 2007)


(3) \begin{eqnarray*}
-c G_0 &=&c \left(\chi _{0P} \frac{4 \pi B}{c}-\chi _{0E} E\right)\left(1+\frac{1}{2}\frac{v^2}{c^2}\right)\\
&&+\, (2\chi _{0E}-\chi _{0F}) \left(\frac{v_i F_i}{c} \right) \\
&&+\, c(\chi _{0F}-\chi _{0E}) \left(\frac{v^2}{c^2}E + \frac{v_i v_j P_{ij}}{c^2}\right),
\end{eqnarray*}

(4) \begin{eqnarray*}
-G_i & =&-\chi _{0F} \frac{{F}_i}{c} \left(1+\frac{1}{2} \frac{v^2}{c^2}\right) + \chi _{0P} \frac{4 \pi B}{c} \frac{v_i}{c} + \chi _{0F} \frac{{v_j} P_{ji}}{c} \\
&& +\, (\chi _{0F}-\chi _{0E}) \left(E-\frac{2 v_j F_j}{c^2}\right) \frac{{v_i}}{c}.
\end{eqnarray*}

Here, a subscript 0 in χ indicates the absorption coefficient is expressed in the comoving rather than the lab frame, and χ_0*P*_, χ_0*E*_, and χ_0*F*_ are the comoving-frame Planck, energy, and flux mean absorption coefficients. The leading-order part of the time-like component of the mixed-frame radiation four-force *G*_0_ is the classical rate of radiation–matter energy exchange in the comoving frame, while the order *v*/*c* part combines the work done by the radiation force on matter (−χ_0*F*_*v_i_F_i_*/*c*) with a purely relativistic effect arising from the transformation of the opacity between the comoving and lab frames (2χ_0*E*_*v_i_F_i_*/*c*); the second-order terms are similarly relativistic effects arising from the frame transformation. In the space-like component *G_i_*, the leading-order term is the radiation force in the comoving frame, while the remaining order *v*/*c* and *v*^2^/*c*^2^ terms describe relativistic effects that can be interpreted as frame-dragging between matter and radiation. Note that it is also possible to evaluate $(cG^0, \boldsymbol{G})$ using the exact Lorentz transformation that is accurate to all orders of *v*/*c* (Mihalas & Auer 2001), and some authors take this approach (Jiang, Stone & Davis 2019; Chang, Davis & Jiang 2020). Our implementation allows users to choose which approach – order *v*/*c*, order *v*^2^/*c*^2^, or exact – to use, but unless stated otherwise in what follows we use order *v*^2^/*c*^2^ accuracy. In non-relativistic problems using frequency-integrated radiative transfer this choice makes little difference, and in forthcoming work we show that retaining only the *v*/*c* terms offers a significant computational advantage in multigroup problems (He, Wibking & Krumholz, in preparation).

### 2.2 Characteristic numbers and limiting regimes

We next derive characteristic dimensionless numbers for RHD systems and consider the limiting behaviour of various terms as we alter their relative sizes. We non-dimensionalize equation (1) and equation (2) following Lowrie, Morel & Hittinger (1999). For the matter quantities, we let ℓ be the characteristic size of the system, *a*_∞_ be the characteristic isothermal sound speed, and ρ_∞_ be the characteristic density, and define


(5) \begin{eqnarray*}
\hat{x}_i = x_i/\ell , \quad \hat{t}= t/(\ell /a_\infty), \quad \hat{\rho }= \rho /\rho _\infty , \quad \hat{v}_i = {v_i}/a_\infty ,
\end{eqnarray*}

(6) \begin{eqnarray*}
\hat{p}= p/(\rho _\infty a_\infty ^2), \quad \hat{T}= T/T_\infty , \quad \hat{E}_{\rm gas} = E_{\rm gas}/(\rho _\infty a_{\infty }^2),
\end{eqnarray*}

Here, the quantities with carets are dimensionless versions of the dimensional quantities. For the radiation quantities, we let *T*_∞_ be the reference temperature and λ_∞_ be the reference length scale^2^, and we define


(7) \begin{eqnarray*}
\hat{E}= E/(a_r T_{\infty }^4), \quad \hat{F}= F/(c a_r T_{\infty }^4), \quad \hat{P}_{ij} = P_{ij}/(a_r T_{\infty }^4)
\end{eqnarray*}

(8) \begin{eqnarray*}
\hat{\chi }_0 = \chi _0 \lambda _\infty , \quad 4 \pi \hat{B}= 4 \pi B/(c a_r T_\infty ^4) = \hat{T}^4,
\end{eqnarray*}

With these definitions, the radiation four-force becomes


(9) \begin{eqnarray*}
-cG_0 &=& \frac{c a_r T_\infty ^4}{\lambda _\infty } \left[ \left(\hat{\chi }_{0P} \hat{T}^4-\hat{\chi }_{0E} \hat{E}\right)\left(1 + \frac{1}{2} \frac{\hat{v}^2}{\mathcal {C}^2}\right) \right. \\
&& \left. +\, \left(2\hat{\chi }_{0E}-\hat{\chi }_{0F}\right) \frac{\hat{v}_i \hat{F}_i}{\mathcal {C}} + \left(\hat{\chi }_{0F}-\hat{\chi }_{0E}\right) \frac{\hat{v}^2 \hat{E}+ \hat{v}_i \hat{v}_j \hat{P}_{ij}}{\mathcal {C}^2} \right]\\
\end{eqnarray*}

(10) \begin{eqnarray*}
\quad \equiv \frac{c a_r T_\infty ^4}{\lambda _\infty } (-\hat{G}_0)
\end{eqnarray*}

(11) \begin{eqnarray*}
-G_i & =& \frac{a_r T_\infty ^4}{\lambda _\infty } \left[ -\hat{\chi }_{0F} \hat{F}_i \left(1 + \frac{1}{2} \frac{\hat{v}^2}{\mathcal {C}^2}\right) + \left(\hat{\chi }_{0P} \hat{T}^4-\hat{\chi }_{0E} \hat{E}\right) \frac{\hat{v}_i}{\mathcal {C}} \right. \\
&& \left. +\, \frac{\hat{\chi }_{0F}}{\mathcal {C}} \left(\hat{v}_j \hat{P}_{ji} + \hat{v}_i \hat{E}\right)-\frac{2}{\mathcal {C}^2}\left(\hat{\chi }_{0F}-\hat{\chi }_{0E}\right) \hat{v}_j \hat{F}_j \hat{v}_i \right]
\end{eqnarray*}

(12) \begin{eqnarray*}
\quad \equiv \frac{a_r T_\infty ^4}{\lambda _\infty } (-\hat{G}_i),
\end{eqnarray*}

and the corresponding non-dimensionalized version of equation (1) is


(13) \begin{eqnarray*}
\frac{\partial }{\partial \hat{t}} {\hat{\boldsymbol{U}}}+ \hat{\nabla }\cdot \hat{\boldsymbol{F}}_{\hat{\boldsymbol{U}}}= \hat{\boldsymbol{S}}_{{\hat{\boldsymbol{U}}}},
\end{eqnarray*}

where $\hat{\nabla }\equiv \ell \nabla$ and


(14) \begin{eqnarray*}
{\hat{\boldsymbol{U}}}= \begin{bmatrix}\hat{\rho }\\
\hat{\rho }\hat{\boldsymbol{v}}\\
\hat{E}_{\rm gas}\\
\hat{E}\\
\hat{\boldsymbol{F}}\end{bmatrix}, \, \hat{\boldsymbol{F}}_{\hat{\boldsymbol{U}}}= \begin{bmatrix}\hat{\rho }\hat{v}\\
\hat{\rho }\left(\hat{\boldsymbol{v}}\otimes \hat{\boldsymbol{v}}+ \hat{T}\right) \\
\left(\hat{E}_{\rm gas} + \hat{p}\right) \hat{\boldsymbol{v}}\\
\mathcal {C}\hat{\boldsymbol{F}}\\
\mathcal {C}\hat{\mathsf {P}}\end{bmatrix}, \, \hat{\boldsymbol{S}}_{{\hat{\boldsymbol{U}}}} = \mathcal {L}\begin{bmatrix}0 \\
\mathcal {P}\hat{\boldsymbol{G}}\\
\mathcal {P}\mathcal {C}\hat{G}_0 \\
-\mathcal {C}\hat{G}_0 \\
-\mathcal {C}\hat{\boldsymbol{G}}\end{bmatrix}. \\
\end{eqnarray*}

In these expressions, we have introduced three dimensionless quantities:


(15) \begin{eqnarray*}
\mathcal {C}= \frac{c}{a_\infty }, \qquad \mathcal {L}= \frac{\ell }{\lambda _\infty }, \qquad \mathcal {P}= \frac{a_r T_\infty ^4}{\rho _\infty a_\infty ^2}.
\end{eqnarray*}

These numbers represent, respectively, the dimensionless speed of light, the ratio of the system size to the photon mean-free path (and thus is equal to the characteristic optical depth), and (up to factors of order unity) the ratio of radiation pressure to gas pressure at the characteristic temperature and sound speed of the system. It is therefore clear that an RHD system is determined by these three characteristic numbers; the first is always much greater than unity for a non-relativistic system, but the remaining two can be of any size.

Now let us consider various limiting cases of the dimensionless numbers, focusing in particular on the evolution equations for the radiation quantities $\hat{E}$ and $\hat{\boldsymbol{F}}$ (the last two entries in equation 13); this will simplify our task since $\mathcal {P}$ does not appear in these equations. For numerical convenience, and since our goal here is insight rather than rigorous calculation, we will also at this point specialize to the case of grey material, which allows us to choose our scaling λ_∞_ such that $\hat{\chi }_{0P} = \hat{\chi }_{0E} = \hat{\chi }_{0F} = 1$. In this case the non-dimensional radiation four-force reduces to


(16) \begin{eqnarray*}
-\hat{G}_0 = \left(\hat{T}^4-\hat{E}\right)\left(1 + \frac{1}{2} \frac{\hat{v}^2}{\mathcal {C}^2}\right) + \frac{\hat{v}_i \hat{F}_i}{\mathcal {C}}
\end{eqnarray*}

(17) \begin{eqnarray*}
-\hat{G}_i = -\hat{F}_i \left(1 + \frac{1}{2} \frac{\hat{v}^2}{\mathcal {C}^2}\right) + \hat{T}^4 \hat{v}_i + \frac{\hat{v}_j \hat{P}_{ji}}{\mathcal {C}}.
\end{eqnarray*}

For $\mathcal {L}\ll 1$, the system is optically thin and we are in the streaming regime. In this case it is clear that the largest term is $\hat{\nabla }\cdot \hat{\boldsymbol{F}}_{{\hat{\boldsymbol{U}}}}$, and so on a fluid flow time-scale it is clear that the solution is simply $\hat{\nabla }\cdot \hat{\boldsymbol{F}}= 0$ and $\hat{\nabla }\cdot \hat{\mathsf {\mathit{ P}}}= \boldsymbol{0}$. On the other hand, for $\mathcal {L}\gg 1$, we are in the diffusion limit, and it is clear that $\hat{\boldsymbol{S}}_{{\hat{\boldsymbol{U}}}}$, which is of order $\mathcal {C}\mathcal {L}$, is the largest term, and therefore on a fluid flow time-scale to leading order we must have $(\hat{G}_0, \hat{\boldsymbol{G}}) = (0, \boldsymbol{0})$. This in turn requires that, to leading order, $\hat{E}= \hat{T}^4$ and $\hat{\boldsymbol{F}}= \boldsymbol{0}$; though we have not shown it here, it is straightforward to show that in this limit we also have $\hat{\mathsf {\mathit{ P}}}= (1/3)\mathsf {I}\hat{E}$, where $\mathsf {I}$ is the identity tensor (Mihalas & Mihalas 1984). Since the flux is zero to the leading order in this case, we must proceed to the next order to determine its value. To do so, we Taylor expand the radiation energy density, flux, and pressure tensor about the leading order solution:


(18) \begin{eqnarray*}
\hat{E}= \hat{T}^4 + \hat{E}_{(1)} \quad \hat{\boldsymbol{F}}= \hat{\boldsymbol{F}}_{(1)} \quad \hat{\mathsf {\mathit{ P}}}= \frac{1}{3}\mathsf {I}\hat{T}^4 + \hat{\mathsf {\mathit{ P}}}_{(1)},
\end{eqnarray*}

where terms subscripted (1) are perturbations that we take to be small compared to the leading-order terms. We insert these expressions into equation (13), Taylor expand, and linearize by dropping all terms that involve products of perturbed quantities. Which terms are at leading order after this procedure depends on the relative values of $\mathcal {C}$ and $\mathcal {L}$. If $\mathcal {C}\gg \mathcal {L}$, known as the static diffusion limit, then the leading order surviving terms in the equation for the flux are


(19) \begin{eqnarray*}
\frac{\partial }{\partial t}\hat{\boldsymbol{F}}_{(1)} + \frac{1}{3} \mathcal {C}\hat{\nabla }\hat{T}^4 = -\mathcal {C}\mathcal {L}\hat{\boldsymbol{F}}_{(1)}.
\end{eqnarray*}

Since both $\mathcal {C}$ and $\mathcal {C}\mathcal {L}$ are large compared to unity, on a fluid flow time-scale the terms proportional to these factors must cancel, and therefore we have


(20) \begin{eqnarray*}
\hat{\boldsymbol{F}}_{(1)} = -\frac{1}{3\mathcal {L}}\hat{\nabla }\hat{T}^4.
\end{eqnarray*}

This is the usual Fick’s Law diffusion approximation. Armed with this leading order result, we can see that the relative sizes of the terms in the radiation evolution equations $(\partial /\partial \hat{t})(\hat{E},\hat{\boldsymbol{F}}): \mathcal {C}\hat{\nabla }\cdot (\hat{\boldsymbol{F}},\hat{\mathsf {P}}): \mathcal {C}\mathcal {L}(\hat{G}_0, \hat{\boldsymbol{G}})$ scale relative to one another as $1 : \mathcal {C}/\mathcal {L}: \mathcal {C}/\mathcal {L}$.

On the other hand, if we have $\mathcal {C}\ll \mathcal {L}$, known as the dynamic diffusion case, then the leading non-zero terms are


(21) \begin{eqnarray*}
\frac{\partial }{\partial t}\hat{\boldsymbol{F}}_{(1)} = -\mathcal {C}\mathcal {L}\hat{\chi }_0 \hat{\boldsymbol{F}}_{(1)} + \frac{4}{3}\mathcal {L}\hat{T}^4 \hat{\boldsymbol{v}}.
\end{eqnarray*}

As in the previous case, since $\mathcal {C}$ and $\mathcal {C}\mathcal {L}$ are large compared to unity, on a fluid flow time-scale the two terms on the right-hand side must cancel to leading order, and we instead have


(22) \begin{eqnarray*}
\boldsymbol{F}_{(1)} = \frac{4}{3\mathcal {C}} \hat{\boldsymbol{v}}\hat{T}^4.
\end{eqnarray*}

In this case the relative scalings of the terms in the radiation evolution equations $(\partial /\partial \hat{t})(\hat{E},\hat{\boldsymbol{F}}): \mathcal {C}\hat{\nabla }\cdot (\hat{\boldsymbol{F}},\hat{\mathsf {\mathit{ P}}}): \mathcal {C}\mathcal {L}(\hat{G}_0, \hat{\boldsymbol{G}})$ are 1: 1: 1, i.e. all terms are of equal order.

### 2.3 Design considerations for numerical methods

While the exploration of the limiting cases here is not new (e.g. Mihalas & Mihalas 1984; Lowrie et al. 1999; Krumholz et al. 2007), revisiting it allows us to make some important observations about design considerations for numerical methods. First, which terms are large on a fluid flow time-scale, and relative to each other, changes from one RHD regime to another – in the streaming limit the transport terms proportional to $\mathcal {C}$ dominate, in the static diffusion regime these terms come into balance with the source terms and both are at order $\mathcal {C}/\mathcal {L}\gg 1$, while in the dynamic diffusion regime all terms, including the time derivative, become of order unity. Therefore if an RHD scheme is to correctly capture the limiting behaviour in each of these regimes, it cannot rely on any particular assumptions about the relative orderings of these terms, and must be able to cope with situations where each of them is both dominant and subdominant.

A second consideration, which we have already introduced in Section 1, is that in the diffusion regime obtaining the correct solution depends on the dominant terms cancelling to high-order. That is, we have seen that the source terms are naturally of size $\mathcal {C}\mathcal {L}$, but in static diffusion the leading order terms cancel and so the dominant non-vanishing term is smaller by a factor $\mathcal {L}^2 \gg 1$, while for dynamic diffusion it is smaller by a factor $\mathcal {C}\mathcal {L}\gg 1$. Similarly, the natural sizes of the transport terms are $\mathcal {C}$, but this is reduced by a factor $\mathcal {L}$ in static diffusion, and by a factor $\mathcal {C}$ in dynamic diffusion. This creates problems for schemes where the transport and source terms, or parts of the source terms, are operator-split, because in an operator split scheme it is difficult to recover the proper near-exact cancellations between the various terms. Overcoming this problem will be our primary objective.

Before describing our proposed solution, however, we pause to discuss another possible approach that is somewhat close to ours in spirit: the DG-IMEX method of McClarren et al. (2008). This method uses a temporal discretization that accurately captures cancellations in the static diffusion limit while avoiding non-local implicit solves, and McClarren et al. show that it passes a number of tests that other operator-split methods fail. However, the price for this is high: the method requires a careful and complex reconstruction scheme that is heavy in terms of both computation and memory usage – indeed, the method requires eight degrees of freedom per cell, and therefore requires eight times as much memory as the finite volume scheme we present below, a major problem for GPU-based computations where memory is at a premium. Additionally, in the full radiation-hydrodynamics context (not considered by McClarren et al.), coupling a DG radiation solver to a finite volume hydrodynamics code requires careful consideration of how the fluid internal energy is mapped from the finite volume grid to the DG nodes in order to maintain the asymptotic-preserving property. While existing methods for this mapping numerically manifest the correct asymptotic diffusion solution, they have not been subjected to a rigorous asymptotic analysis such as the one we present below for our scheme, and thus their ability to produce correct asymptotic behaviour over all parameter regimes remains unproven (Bolding et al. 2017). Moreover, extending a DG scheme to adaptive mesh refinement (AMR) would be a substantial challenge, whereas the finite volume scheme we propose is fully compatible with existing AMR frameworks. Finally, McClarren et al. focus exclusively on the static diffusion regime, and their scheme is not easily extensible to either the streaming or dynamic diffusion limits – the former because the method relies on a specific closure relation that is a poor approximation for streaming radiation, and the latter because the scheme does not include the velocity-dependent terms that become order unity in the dynamic diffusion limit. Our scheme, by contrast, applies to all RHD regimes.

## 3 A NEW ASYMPTOTIC-PRESERVING SCHEME FOR RADIATION HYDRODYNAMICS

We now proceed to describe our new numerical method. We first describe our overall strategy for the full RHD system in Section 3.1, then the IMEX scheme we use for the radiation subsystem in Section 3.2, and finally our method for carrying out each IMEX stage in Section 3.3.

### 3.1 Overall time stepping strategy

We solve the system formed by equation (1) using an operator split approach consisting of two major steps. In the first step, we advance the hydrodynamic transport subsystem using an explicit method. In the second step, we update the radiation transport subsystem and matter–radiation coupling terms using a mixed IMEX method.

The hydrodynamic transport subsystem consists of the partial differential equations


(23) \begin{eqnarray*}
\frac{\partial }{\partial t} \begin{bmatrix}\rho \\
\rho \boldsymbol{v} \\
E_{\rm gas} \end{bmatrix} + \nabla \cdot \begin{bmatrix}\rho \boldsymbol{v} \\
\rho \boldsymbol{v} \otimes \boldsymbol{v}+p \\
(E_{\rm gas} + p) \boldsymbol{v} \end{bmatrix} = 0.
\end{eqnarray*}

Our scheme for radiation does not depend on the numerical method used to solve this subsystem; for the purposes of our implementation in quokka ([Bibr bib37][Bibr bib37]), which we use for all the tests below, we adopt a semidiscrete approach, discretizing the spatial variables on to a grid while initially keeping the time variable continuous, thereby transforming the partial differential equations into a large set of ordinary differential equations (ODEs). These ODEs are then integrated in time utilizing the second-order accurate, strong stability preserving Runge–Kutta method (RK2-SSP; Shu & Osher 1988). For an in-depth explanation of the method we direct readers to [Bibr bib37][Bibr bib37].

In this work, our emphasis is on the second major step, the radiation update and matter–radiation coupling. These steps are subcycled with respect to the hydrodynamic step, since they require smaller time-steps.

### 3.2 Implicit–explicit method for the radiation subsystem

Using the same method of lines approach for the radiation subsystem and radiation–matter coupling terms as for hydrodynamics, we define *E_ijk_* as the volume-averaged radiation energy density in cell *ijk*, and similarly for all other variables, and express the radiation subsystem for each cell as


(24) \begin{eqnarray*}
\frac{\mathrm{ d}}{\mathrm{ d}t} \boldsymbol{U}_{ijk} = \boldsymbol{T}(\boldsymbol{y}_{ijk}, t) + \boldsymbol{S}(\boldsymbol{y}_{ijk}, t),
\end{eqnarray*}

where, dropping the *ijk* subscript from this point forward for convenience, we define


(25) \begin{eqnarray*}
\boldsymbol{U}= \begin{bmatrix}\rho \boldsymbol{v} \\
E_{\rm gas} \\
E \\
\frac{1}{c^2} \boldsymbol{F} \end{bmatrix}, \quad \boldsymbol{T}= - \nabla \cdot \begin{bmatrix}0 \\
0 \\
\boldsymbol{F} \\
\mathsf {\mathit{ P}}\end{bmatrix}, \quad \boldsymbol{S}= \begin{bmatrix}\boldsymbol{G} \\
c G_0 \\
-c G_0 \\
-\boldsymbol{G} \end{bmatrix},
\end{eqnarray*}

The characteristic time-scales associated with the transport term, $\boldsymbol{T}$, which consists of the divergence of radiation flux, $\nabla \cdot \boldsymbol{F}$, and the divergence of the radiation pressure tensor $\nabla \cdot \mathsf {\mathit{ P}}$, are $\mathcal {C}^{-1}$, which is potentially fast compared to hydrodynamics, but in the diffusion regime is much longer than the time-scale $(\mathcal {C}\mathcal {L})^{-1}$ associated with the source term $\boldsymbol{S}$.^3^ Consequently, we opt to evolve the transport terms using an explicit method. This choice is strategic; the explicit update obviates the need for global communication across the computational domain. This advantage becomes particularly pronounced on GPUs, where interdevice communication often represents the primary bottleneck in performance. In problems where $\mathcal {C}$ is so large that this requires infeasibly many explicit steps, we may elect to solve the approximate RSLA equations instead (Gnedin & Abel 2001; Skinner & Ostriker 2013; Wibking & Krumholz 2022), for which we instead have


(26) \begin{eqnarray*}
\boldsymbol{U}= \begin{bmatrix}\rho \boldsymbol{v} \\
E_{\rm gas} \\
E \\
\frac{1}{c\hat{c}} \boldsymbol{F}_r \end{bmatrix}, \quad \boldsymbol{T}= - \nabla \cdot \begin{bmatrix}0 \\
0 \\
\frac{\hat{c}}{c}\boldsymbol{F} \\
\mathsf {\mathit{ P}}\end{bmatrix}, \quad \boldsymbol{S}= \begin{bmatrix}\boldsymbol{G} \\
c G_0 \\
-\hat{c} G_0 \\
-\boldsymbol{G} \end{bmatrix},
\end{eqnarray*}

where $\hat{c}$ is the reduced speed of light, chosen so to be ≪*c* but still much greater than any hydrodynamic speed. This approximation reduces the size of the transport term from $\mathcal {C}$ to $(\hat{c}/c)\mathcal {C}$, and thus allows larger time-steps. For generality in what follows we will write our update scheme using the RSLA equations, but these can be reduced to the exact equations of RHD simply by setting $\hat{c} = c$.

The short time-scales associated with the matter–radiation coupling terms, $\boldsymbol{S}$, require an implicit treatment. Notably, as these terms do not have spatial derivatives, they allow for the independent update of each cell within the computational domain. Therefore, in the whole radiation update, there is no non-local implicit update, effectively eliminating the requirement for additional interdomain communication beyond what is standard in a pure hydrodynamic update.

To ensure that our choice to operator-split between the source and transport terms in this manner does not compromise accuracy, we utilize the asymptotic-preserving IMEX PD-ARS integrator (Chu et al. 2019), a choice motivated by its proven convex-invariant property^4^ and effectiveness in preserving the diffusion limit while maintaining second-order accuracy and stability in the streaming limit.^5^The IMEX PD-ARS integrator can be characterized by its double Butcher tableau


(27) \begin{eqnarray*}
{\cal B}_{\rm PD-ARS} =
\begin{array}{c|ccc}0 & 0 & 0 & 0 \\
1 & 1 & 0 & 0 \\
1 & 1/2 & 1/2 & 0 \\
\hline & 1/2 & 1/2 & 0 \end{array}\qquad \begin{array}{c|ccc}0 & 0 & 0 & 0 \\
1 & 0 & 1 & 0 \\
1 & 0 & 1/2-\epsilon & 1/2 + \epsilon \\
\hline & 0 & 1/2-\epsilon & 1/2 + \epsilon , \end{array}
\end{eqnarray*}

where ϵ is a free parameter in the range [0,0.5); in our implementation we adopt ϵ = 0. When expressed in equations, the scheme to advance the system by a time Δ*t* is


(28) \begin{eqnarray*}
\boldsymbol{U}^{(n+1/2)} = ~\boldsymbol{U}^{(n)} + \Delta t ~\boldsymbol{T}(\boldsymbol{U}^{(n)}) + \Delta t ~\boldsymbol{S}(\boldsymbol{U}^{(n+1/2)})
\end{eqnarray*}

(29) \begin{eqnarray*}
\boldsymbol{U}^{(n+1)} &=& ~\boldsymbol{U}^{(n)} + \Delta t \left[\frac{1}{2} ~\boldsymbol{T}(\boldsymbol{U}^{(n)}) + \frac{1}{2} ~\boldsymbol{T}(\boldsymbol{U}^{(n+1/2)}) \right] \\
&&+\, \Delta t \left[\frac{1}{2} ~\boldsymbol{S}(\boldsymbol{U}^{(n+1/2)}) + \frac{1}{2} ~\boldsymbol{S}(\boldsymbol{U}^{(n+1)}) \right],
\end{eqnarray*}

where the superscript (*n*) indicates the state at the start of the radiation update (but after the operator-split hydrodynamic update), (*n* + 1) indicates the state at the end of the radiation update, and (*n* + 1/2) indicates an intermediate stage.

One key feature of this update scheme is that the transport and source terms appear symmetrically at each of the two stages, so that cancellations can be captured properly. A second key feature is that, on modern architectures where communication is expensive, this scheme is only marginally more costly than an update like RK2-SSP, because the only excess work it requires is an extra iterative solve at the first stage (equation 28). Crucially, this extra solve is purely local, because only the local source terms, rather than the non-local transport terms, must be iterated. Thus, there is no extra communication in this scheme relative to RK2-SSP.

### 3.3 Update strategy for IMEX stages

At each stage of the integrator above, the right-hand side contains both explicit terms – those that are evaluated at state (*n*) during the first IMEX stage (equation 28) and at states (*n*) or (*n* + 1/2) during the second stage (equation 29) and implicit terms that are evaluated at state (*n* + 1/2) during the first stage and state (*n* + 1) during the second stage. We therefore begin each stage by evaluating the explicit terms. This in turn requires that we evaluate the transport terms $\boldsymbol{T}$, which are always explicit in keeping with our overall strategy. We do this using a Godunov method. Specifically, we compute the fluxes for *E* and $\boldsymbol{F}$ between cells by using Piecewise Parabolic Method (PPM) reconstruction to obtain the states at the cell edges and then using a Harten, Lax, van Leer (HLL) Riemann solver (Harten, Lax & van Leer 1983) to compute the fluxes from these states. The details of this step are elaborated in [Bibr bib37][Bibr bib37], and hence we will not repeat them here. However, we modify the scheme in an important way compared to the method presented in [Bibr bib37][Bibr bib37]: that scheme, and all previous comparable explicit treatments of radiation, have been forced to invoke an ad hoc correction to the wavespeeds computed in the Riemann solver in an attempt to recover the diffusion limit. With our new time-stepping scheme, no such modification is required, and we can simply use the uncorrected HLL fluxes in our update. We discuss this issue further in Section 4.3.

After carrying out the explicit part of the update, we are left with the implicit part. For both stages of the IMEX integrator we can express this stage in the generic form


(30) \begin{eqnarray*}
E_{\rm gas}^{(t+1)}-E_{\rm gas}^{(t)} = c G_0^{(t+1)} \theta \Delta t
\end{eqnarray*}

(31) \begin{eqnarray*}
E^{(t+1)}-E^{(t)} = - \hat{c}G_0^{(t+1)} \theta \Delta t
\end{eqnarray*}

for the energies, and


(32) \begin{eqnarray*}
(\rho \boldsymbol{v})^{(t+1)}-(\rho \boldsymbol{v})^{(t)} = \boldsymbol{G}^{(t+1)} \theta \Delta t
\end{eqnarray*}

(33) \begin{eqnarray*}
\boldsymbol{F}^{(t+1)}-\boldsymbol{F}^{(t)} = - \hat{c}c \boldsymbol{G}^{(t+1)} \theta \Delta t
\end{eqnarray*}

for the momenta. Here, terms with superscript (*t*) denote the state after applying the explicit terms – for example during the first stage (equation 28) we have $E^{(t)} = E^{(n)}-\Delta t (\hat{c}/c) \nabla \cdot \boldsymbol{F}^{(n)}$ – and terms with superscript (*t* + 1) denote the final state for which we are attempting to solve; the factor θ is unity during the first stage and 1/2 during the second stage.

In each cell this is a system of eight equations in eight unknowns – radiation and gas energy, three components of gas velocity, and three components of radiation flux – that must be solved simultaneously. While we could do so straightforwardly using a single Newton–Raphson iteration scheme or similar, it is more efficient to separate the iteration procedure into an inner stage where we solve the energy equations while freezing $\boldsymbol{v}$ and $\boldsymbol{F}$, and a second, outer stage where update the flux and gas velocity and then if necessary go back to the inner stage and recompute *E* and *E*_gas_ using the updated values of $\boldsymbol{v}$ and $\boldsymbol{F}$. The reason this is more efficient is that it allows the inner iteration stage to consider only two variables rather than eight, and in most cases the work terms proportional to $\boldsymbol{v}$ and $\boldsymbol{F}$ are small in the energy equations, so *E* and *E*_gas_ change little to none as a result of the update to $\boldsymbol{v}$ and $\boldsymbol{F}$ and the whole procedure converges in a single or at most a few outer iterations.

The inner stage consists of updating the energy quantities using a modified version of the iteration scheme initially proposed by Howell & Greenough (2003) and modified by [Bibr bib37][Bibr bib37]. We solve equation (30) and equation (31) for $E_{\rm gas}^{(t+1)}$ and *E*^(*t* + 1)^ by performing Newton–Raphson iteration on the non-linear implicit system


(34) \begin{eqnarray*}
0 = F_G(E_{\rm gas}^{(t+1)}, E^{(t+1)}) \equiv E_{\rm gas}^{(t+1)}-E_{\rm gas}^{(t)} + \left(\frac{c}{\hat{c}} \right) R^{(t+1)},
\end{eqnarray*}

(35) \begin{eqnarray*}
0 = F_R(E_{\rm gas}^{(t+1)}, E^{(t+1)}) \equiv E^{(t+1)}-E^{(t)}-(R^{(t+1)} + S^{(t+1)}),
\end{eqnarray*}

where


(36) \begin{eqnarray*}
R^{(t+1)} &\equiv& -\hat{c}G_0^{(t+1)} \theta \Delta t \\
&=& \theta \Delta t \left[\hat{c}\left(\chi _{0P} \frac{4 \pi B}{c}-\chi _{0E} E\right)\left(1 + \frac{1}{2} \frac{v^2}{c^2}\right)\right. \\
&& +\, (2\chi _{0E}-\chi _{0F}) \frac{\hat{c}}{c} \left(\frac{v_i F_i}{c} \right) \\
&&\left. +\, \hat{c} (\chi _{0F}-\chi _{0E}) \left(\frac{v^2}{c^2}E + \frac{v_i v_j P_{ij}}{c^2}\right)\right],
\end{eqnarray*}

and *S* is an optional term to include, for example, the addition of radiation by stellar sources. Note that *E*, $\mathsf {\mathit{ P}}$, and all the variables that can depend on *E*_gas_ – χ_0*P*_, χ_0*E*_, χ_0*F*_, and *B* – carry the superscripts (*t* + 1) which are omitted here for the sake of brevity. By contrast, we freeze $\boldsymbol{v}$ and $\boldsymbol{F}$ at their values at the start of the inner stage as noted above; thus these terms do not carry subscripts (*t* + 1), and do not evolve during the inner Newton–Raphson stage.

A single Newton–Raphson iteration consists of solving the linearized equations


(37) \begin{eqnarray*}
\mathbf {J} \cdot \Delta \boldsymbol{x}= - \boldsymbol{F}(\boldsymbol{x}),
\end{eqnarray*}

where $\boldsymbol{x}$ is the set of variables to be updated, $\Delta \boldsymbol{x}$ is the change in these variables during this iteration, $\boldsymbol{F}(\boldsymbol{x})$ is the vector whose zero we wish to find, and **J** is the Jacobian matrix of $\boldsymbol{F}(\boldsymbol{x})$. Instead of taking $\boldsymbol{x}= (E_{\rm gas}, E)$, as in the original Howell & Greenough (2003) scheme, we use $\boldsymbol{x}= (E_{\rm gas}, R)$ as the base variables over which to iterate; we find that the system generally converges in fewer iterations using this basis, likely because at high-optical depth the system almost immediately converges to the solution *R* = 0, and thus the remaining iterations are effectively on *E*_gas_ alone The Jacobian in this basis is


(38) \begin{eqnarray*}
\frac{\partial F_G}{\partial E_{\rm gas}} &=& 1 \\
\frac{\partial F_G}{\partial R} &=& \frac{c}{\hat{c}} \\
\frac{\partial F_{R}}{\partial E_{\rm gas}} &=& \left.\frac{\partial E}{\partial E_{\rm gas}}\right|_{R = {\rm const.}} = \frac{1}{C_v} \frac{\partial }{\partial T} \left(\frac{\chi _{0P}}{\chi _{0E}} \frac{4 \pi B}{c} \right) \\
\frac{\partial F_{R}}{\partial R} &=& \left.\frac{\partial E}{\partial R}\right|_{T = {\rm const.}}-1 = - \frac{1}{\hat{c} \ \chi _{0E} \ \theta \Delta t }-1.
\end{eqnarray*}

We have omitted the *v*^2^/*c*^2^ terms and assumed $\partial (\kappa _P/\kappa _E)/\partial T = 0$ in the calculation of the Jacobian for simplicity, but this simplification only changes the rate of convergence; it does not affect the converged solution.

After solving equation (37) for $\Delta \boldsymbol{x}$, we update $\boldsymbol{x}\leftarrow \boldsymbol{x}+ \Delta \boldsymbol{x}$; we do this repeatedly until the system converges, as determined by the condition


(39) \begin{eqnarray*}
\left| \frac{F_G}{E_{\rm tot}} \right| < \epsilon \quad {\rm and} \quad \left| \frac{c}{\hat{c}} \frac{F_R}{E_{\rm tot}} \right| < \epsilon
\end{eqnarray*}

where


(40) \begin{eqnarray*}
E_{\rm tot} = E_{\rm gas}^{(t)} + \frac{\hat{c}}{c} (E_r^{(t)} + S^{(t)}).
\end{eqnarray*}

is the total radiation and material energy at the beginning of the time-step accounted for reduced speed of light. We set the relative tolerance ϵ = 10^−13^ by default. Once this Newton–Raphson system converges, we have the updated gas total energy $E_{\rm gas}^{(t+1)}$ and we can compute the updated radiation energy as


(41) \begin{eqnarray*}
E^{(t+1)} = E^{(t)}-\frac{c}{\hat{c}}(E_{\rm gas}^{(t+1)}-E_{\rm gas}^{(t)}) + S^{(t+1)}.
\end{eqnarray*}

Note that, for $\hat{c} = c$ and *S* = 0, this procedure ensures that our scheme conserves total energy to machine precision regardless of the level of accuracy with which we have iterated the equations to convergence.

We then proceed to the outer stage of the iteration where we solve the flux and momentum update equations, equation (32) and equation (33), with the updated gas temperature, opacity, and radiation energy. To order *v*/*c*, the solution is straightforward:


(42) \begin{eqnarray*}
F_i^{(t+1)} = \frac{F_i^{(t)} + \hat{c} \theta \Delta t\left[\chi _{0P} \frac{4 \pi B}{c} v_i {+} \chi _{0F} v_j P_{ji} {+} (\chi _{0F}{-}\chi _{0E}) E v_i \right]}{1 + \hat{c}\chi _{0F} \theta \, \Delta t}.\\
\end{eqnarray*}

To order *v*^2^/*c*^2^, in cases where χ_0*F*_ − χ_0*E*_ = 0, the solution is


(43) \begin{eqnarray*}
F_i^{(t+1)} = \frac{F_i^{(t)} + \hat{c} \theta \Delta t \left(\chi _{0P} \frac{4 \pi B}{c} v_i + \chi _{0F} v_j P_{ji} \right)}{1 + \hat{c}\chi _{0F} (1 + v^2/2c^2) \theta \Delta t }.
\end{eqnarray*}

When χ_0*F*_ ≠ χ_0*E*_ and to order *v*^2^/*c*^2^, the solution is slightly more complex because all three components of *F* appear in *G_i_*. In this case, $F_i^{(n+1)}$ is the solution of a set of three linear equations; these are straightforward to solve analytically, but the resulting expressions are somewhat lengthy and so we omit them here for brevity. Lastly, following equation (32), we update the gas momentum via


(44) \begin{eqnarray*}
(\rho v_i)^{(t+1)} = (\rho v_i)^{(t)}-\frac{F_i^{(t+1)}-F_i^{(t)}}{c \hat{c}}.
\end{eqnarray*}

This update also ensures momentum conservation to machine precision. After we update the gas momentum, we also recalculate the gas’s internal energy (which in quokka we track separately because we implement a dual energy formalism) by subtracting the updated kinetic energy from the updated total energy.

As previously indicated, the gas velocity $\boldsymbol{v}$ and radiation flux $\boldsymbol{F}$ we use in the inner stage of the iteration are lagged. This can cause significant inaccuracies at high-optical depths when the velocity-dependent terms are non-negligible. To eliminate errors like this and render this scheme fully implicit, we now repeat the inner iteration using the updated values of $\boldsymbol{v}$ and $\boldsymbol{F}$, and compute new estimates for $\boldsymbol{v}$ and $\boldsymbol{F}$, repeating this procedure until either the relative change in the value of the terms proportional to $\boldsymbol{v}$ and $\boldsymbol{F}$ in *R* (equation 36) from one outer iteration to the next is below 10^−13^ or the absolute change is below 10^−13^*R*. Except in the dynamic diffusion limit, where the velocity-dependent terms are at the same order as all other terms, this iterative process typically terminates after just one iteration, and in all the tests we present below, and for all the test problems presented in [Bibr bib37][Bibr bib37], we never require more than a handful of outer iterations. Thus the cost is modest. This completes accounting for all terms in the radiation four-force, thus completing radiation–matter coupling.

## 4 PROPERTIES OF THE SCHEME IN THE DIFFUSION LIMIT

Before proceeding to numerical tests of the scheme we have described, we first present an analysis of its behaviour in the so-called asymptotic diffusion regime, where the photon mean-free path is not resolved by the computational grid, in order to demonstrate directly why it succeeds in capturing this limit while other schemes fail.

### 4.1 The asymptotic diffusion limit of the discrete IMEX equations

We begin our analysis by recalling the results from Section 2.2, which are that in the static diffusion limit for grey material with $\hat{\chi }_{0E} = \hat{\chi }_{0F} = \hat{\chi }_{0P} = 1$, to leading order the radiation energy in non-dimensionalized variables is


(45) \begin{eqnarray*}
\hat{E}= \hat{T}^4
\end{eqnarray*}

and the radiation flux is


(46) \begin{eqnarray*}
\hat{\boldsymbol{F}}= - \frac{1}{3 \mathcal {L}} \hat{\nabla }\hat{T}^4.
\end{eqnarray*}

Inserting these limits into the evolution equations for matter and radiation energy that we solve during the radiation update (equation 13; i.e. omitting changes in the matter energy due to fluid processes), we have


(47) \begin{eqnarray*}
\frac{\partial \hat{E}_\mathrm{gas}}{\partial \hat{t}} &= \mathcal {P}\mathcal {C}\hat{G}_0
\end{eqnarray*}

(48) \begin{eqnarray*}
\frac{\partial \hat{E}}{\partial \hat{t}} &= \frac{\mathcal {C}}{3\mathcal {L}} \hat{\nabla }^2 \hat{T}^4-\mathcal {C}\hat{G}_0,
\end{eqnarray*}

and thus the evolution equation for the total matter plus radiation energy reduces to the usual radiation-diffusion form,^6^


(49) \begin{eqnarray*}
\frac{\partial }{\partial \hat{t}}\left(\hat{E}+ \mathcal {P}^{-1}\hat{E}_\mathrm{gas}\right) = \frac{\mathcal {C}}{3\mathcal {L}} \hat{\nabla }^2 \hat{T}^4.
\end{eqnarray*}

For a numerical method for thermal radiative transfer to be ‘asymptotic preserving’, it must give a valid discretization of equation (49) and enforce the conditions in equation (45) and equation (46) when $\mathcal {C}\gg \mathcal {L}\gg 1$. With an asymptotic preserving method, it is possible to use cells that are optically thick and still obtain accurate solutions of radiative transfer.

To verify that our IMEX scheme satisfies this condition, we begin by writing down the two steps of the IMEX update for the radiation energy (equation 28 and equation 29), again using non-dimensionalized variables and assuming grey material. For simplicity we will adopt $\hat{\boldsymbol{v}}= 0$ and $\hat{c} = c$ as well. This gives


(50) \begin{eqnarray*}
\frac{\hat{E}^{(n+1/2)}-\hat{E}^{(n)}}{\Delta \hat{t}}
&=& -\hat{\nabla }\cdot (\mathcal {C}\hat{\boldsymbol{F}}^{(n)})\\
&& -\,{\cal L} \mathcal {C}\left[\hat{E}^{(n+1/2)}-(\hat{T}^{(n+1/2)})^4 \right],
\end{eqnarray*}

followed by


(51) \begin{eqnarray*}
&&\frac{\hat{E}^{(n+1)}-\hat{E}^{(n)}}{\Delta \hat{t}}\\
&&\quad = - \frac{1}{2} \left[\hat{\nabla }
\cdot (\mathcal {C}\hat{\boldsymbol{F}}^{(n)}) + \hat{\nabla }\cdot (\mathcal {C}
\hat{\boldsymbol{F}}^{(n+1/2)}) \right] \\
&&\qquad -\,\frac{1}{2} {\cal L} \mathcal {C}\left[\hat{E}^{(n+1/2)}-(\hat{T}^{(n+1/2)})^4
+ \hat{E}^{(n+1)}-(\hat{T}^{(n)})^4 \right],
\end{eqnarray*}

From equation (50) we can solve for *E*^(*n* + 1/2)^ and expand to first order in ${\cal L}^{-1}$ to obtain


(52) \begin{eqnarray*}
\hat{E}^{(n+1/2)} = (\hat{T}^{(n+1/2)})^4 + {\cal L}^{-1} \left[(\mathcal {C}\Delta \hat{t})^{-1} \hat{E}^{(n)}-\hat{\nabla }\cdot \hat{\boldsymbol{F}}^{(n)} \right].
\end{eqnarray*}

Thus in limit $\mathcal {C}\gg \mathcal {L}\gg 1$, we have $\hat{E}^{(n+1/2)} = (\hat{T}^{(n+1/2)})^4$. Similarly, performing the same operation on equation (51), one can show to leading order in $\mathcal {L}^{-1}$ that


(53) \begin{eqnarray*}
\hat{E}^{(n+1)} = (T^{(n+1)})^4.
\end{eqnarray*}

We have therefore established our scheme enforces equation (45) at both stages of the IMEX update.

We next examine the two IMEX stages for the radiation flux,^7^


(54) \begin{eqnarray*}
\frac{\hat{\boldsymbol{F}}^{(n+1/2)}-\hat{\boldsymbol{F}}^{(n)}}{\Delta \hat{t}} = -\hat{\nabla }\left(\frac{\mathcal {C}E^{(n)}}{3}\right)-{\cal L} \mathcal {C}\hat{\boldsymbol{F}}^{(n+1/2)}
\end{eqnarray*}

(55) \begin{eqnarray*}
\frac{\hat{\boldsymbol{F}}^{(n+1)}-\hat{\boldsymbol{F}}^{(n)}}{\Delta \hat{t}} &=& \frac{1}{2} \left[\hat{\nabla }\left(\frac{\mathcal {C}\hat{E}^{(n)}}{3}\right) + \hat{\nabla }\left(\frac{\mathcal {C}\hat{E}^{(n+1/2)}}{3}\right) \right] \\
&& -\,\frac{1}{2} {\cal L} \mathcal {C}(\hat{\boldsymbol{F}}^{(n+1/2)} + \hat{\boldsymbol{F}}^{(n+1)}).
\end{eqnarray*}

equation (54) implies


(56) \begin{eqnarray*}
\boldsymbol{F}^{(n+1/2)} = - \frac{1}{1 + {\cal L} \mathcal {C}\Delta \hat{t}} \left(\frac{\mathcal {C}\Delta \hat{t}}{3} \hat{\nabla }\hat{E}^{(n)}-\boldsymbol{F}^{(n)} \right)
\end{eqnarray*}

and if we again take the limit $\mathcal {C}\gg \mathcal {L}\gg 1$, to leading order we have


(57) \begin{eqnarray*}
\hat{\boldsymbol{F}}^{(n+1/2)} = - \frac{1}{3\mathcal {L}} \hat{\nabla }\hat{E}^{(n)} = - \frac{1}{3\mathcal {L}} \hat{\nabla }(\hat{T}^{(n)})^4.
\end{eqnarray*}

Applying the same procedure to equation (55) yields leading-order terms


(58) \begin{eqnarray*}
\hat{\boldsymbol{F}}^{(n+1)} = - \frac{1}{3\mathcal {L}}\hat{\nabla }\hat{E}^{(n+1/2)} = - \frac{1}{3\mathcal {L}}\hat{\nabla }(\hat{T}^{(n+1/2)})^4.
\end{eqnarray*}

Thus the leading term of the radiation flux reduces the form given by equation (46), simply lagged by a half-step. This none the less means that our scheme obeys this constraint.

Finally, we write down the two IMEX stages for the matter energy update


(59) \begin{eqnarray*}
\mathcal {P}^{-1} \frac{\hat{E}_{\rm gas}^{(n+1/2)}-\hat{E}_{\rm gas}^{(n)}}{\Delta \hat{t}} &= {\cal L} \mathcal {C}\left[\hat{E}^{(n+1/2)}-(\hat{T}^{(n+1/2)})^4 \right],
\end{eqnarray*}

(60) \begin{eqnarray*}
\mathcal {P}^{-1} \frac{\hat{E}_{\rm gas}^{(n+1)}-\hat{E}_{\rm gas}^{(n)}}{\Delta \hat{t}}
&=& \frac{1}{2} {\cal L} \mathcal {C}\left[\hat{E}^{(n+1/2)}-(\hat{T}^{(n+1/2)})^4 \right. \\
&& \left. +\, E^{(n+1)}-(T^{(n)})^4 \right],
\end{eqnarray*}

Adding equation (50) to equation (59) and using equation (58) we get


(61) \begin{eqnarray*}
&& \frac{\hat{E}^{(n+1/2)}-\hat{E}^{(n)}}{\Delta \hat{t}} + \mathcal {P}^{-1} \frac{\hat{E}_{\rm gas}^{(n+1/2)}-\hat{E}_{\rm gas}^{(n)}}{\Delta \hat{t}} \\
&& \quad = - \hat{\nabla }\cdot (\mathcal {C}\hat{\boldsymbol{F}}^{(n)}) \\
&& \quad = \frac{\mathcal {C}}{3\mathcal {L}} \hat{\nabla }^2 (\hat{T}^{(n-1/2)})^4,
\end{eqnarray*}

where the superscript (*n* − 1/2) indicates the intermediate state of the *previous* time-step. Similarly, adding equation (51) to equation (60) and using equation (57) and equation (58) gives


(62) \begin{eqnarray*}
&& \frac{\hat{E}^{(n+1)}-\hat{E}^{(n)}}{\Delta \hat{t}} + \mathcal {P}^{-1} \frac{\hat{E}_{\rm gas}^{(n+1)}-\hat{E}_{\rm gas}^{(n)}}{\Delta \hat{t}} \\
&&\quad = - \frac{1}{2} \left(\hat{\nabla }\cdot (\mathcal {C}\hat{\boldsymbol{F}}^{(n)}) + \hat{\nabla }\cdot (\mathcal {C}\hat{\boldsymbol{F}}^{(n+1/2)}) \right) \\
&&\quad = \frac{\mathcal {C}}{3\mathcal {L}} \left(\frac{1}{2}\right) \left[\hat{\nabla }^2(\hat{T}^{(n-1/2)})^4 + \hat{\nabla }^2 (\hat{T}^{(n)})^4 \right].
\end{eqnarray*}

The combination of equation (61) and equation (62) represents a valid discretization of the diffusion equation (49), albeit one using a temperature that is lagged by half a step, thus proving our IMEX PD-ARS scheme preserves the asymptotic diffusion limit.

It is worth noting that schemes such as the one described by equation (61) and equation (62), while they represent valid discretizations of the diffusion equation, can be unstable depending on how the spatial derivatives are evaluated. In particular, Radice et al. (2018) point out that, in a scheme where computation of $\hat{\boldsymbol{F}}$ in the diffusion limit effectively reduces to computing a centred difference on $\hat{E}$, and in turn evaluating $\hat{\nabla }\cdot \hat{\boldsymbol{F}}$ in the equations above reduces to evaluating a centred difference on $\hat{\boldsymbol{F}}$, the resulting scheme has the property that the solutions in even- and odd-numbered cells are decoupled, i.e. the solution in cell *i* depends only on the states in cells *i* − 2 and *i* + 2, not *i* − 1 and *i* + 1; this in turn can give rise to an even–odd instability where numerical oscillations with a period of two cells are not damped and can grow large. Radice et al. propose a method to suppress this instability. In our tests, while we observe faint hints of the instability in our scheme, these appear only in the dynamic diffusion regime and only in tests at very low resolution (e.g. <64 cells per linear dimension). In all other problems the instability, if it exists at all, is imperceptibly small. We therefore do not use Radice et al.’s correction for any of the tests we present below. However, we have implemented it in quokka, and allow users to enable it via a compile-time option should it prove useful at some point in the future.

### 4.2 Comparison to schemes with purely explicit intermediate stages

It is instructive at this point to repeat the analysis we have just performed for the IMEX discretization for the RK2-SSP discretization used in [Bibr bib37][Bibr bib37], since this will let us see why this scheme does not successfully capture the asymptotic diffusion regime. While our analysis will be specific to this particular time stepping approach, we will see that the results generalize straightforwardly to any scheme where the intermediate stage is fully explicit and includes only the transport terms, and thus to other schemes such as those proposed by Rosdahl & Teyssier (2015) and Skinner et al. (2019).

The RK2-SSP scheme uses the time update


(63) \begin{eqnarray*}
\boldsymbol{\boldsymbol{U}}^{(n+1/2)} = \boldsymbol{\boldsymbol{U}}^{(n)} &+ \Delta t ~\boldsymbol{T}(\boldsymbol{U}^{(n)})
\end{eqnarray*}

(64) \begin{eqnarray*}
\boldsymbol{U}^{(n+1)} &=& \boldsymbol{U}^{(n)} + \Delta t \left[\frac{1}{2} ~\boldsymbol{T}(\boldsymbol{U}^{(n)}) + \frac{1}{2} ~\boldsymbol{T}(\boldsymbol{U}^{(n+1/2)}) \right] \\
&&+\, \Delta t \boldsymbol{S}(\boldsymbol{U}^{(n+1)})
\end{eqnarray*}

Compared to equation (28) and equation (29), the primary difference is in the treatment of the source terms $\boldsymbol{S}$; in the IMEX scheme these appear at both stages of the update, while in the RK2-SSP scheme they appear only at the final stage. This will prove to be crucial in what follows.

Again adopting the limit $\mathcal {C}\gg \mathcal {L}\gg 1$ and setting $\mathsf {\mathit{ P}}= (1/3) \mathsf {I}E$, as appropriate for static diffusion, we can write down the leading-order terms in the two stages of this update for the radiation energy as


(65) \begin{eqnarray*}
\hat{E}^{(n+1/2)} = \hat{E}^{(n)}-\mathcal {C}\Delta \hat{t}\hat{\nabla }\cdot \hat{\boldsymbol{F}}^{(n)}
\end{eqnarray*}

(66) \begin{eqnarray*}
\hat{E}^{(n+1)} = (\hat{T}^{(n+1)})^4.
\end{eqnarray*}

For the radiation flux, we obtain


(67) \begin{eqnarray*}
\hat{\boldsymbol{F}}^{(n+1/2)} = \hat{\boldsymbol{F}}^{(n)}-\frac{\mathcal {C}\Delta \hat{t}}{3} \hat{\nabla }\hat{E}^{(n)},
\end{eqnarray*}

(68) \begin{eqnarray*}
\hat{\boldsymbol{F}}^{(n+1)} = -\frac{1}{3\mathcal {L}} \left(\frac{1}{2}\right)\left(\hat{\nabla }\hat{E}^{(n)} + \hat{\nabla }\hat{E}^{(n+1/2)}\right).
\end{eqnarray*}

Finally, if we write down the update for the gas energy (which is non-trivial only for the second stage, since $\boldsymbol{T}= 0$ for the gas energy) and add it to that for the radiation energy,


(69) \begin{eqnarray*}
&& \frac{\hat{E}^{(n+1)}-\hat{E}^{(n)}}{\Delta \hat{t}} + \mathcal {P}^{-1}\frac{\hat{E}_{\rm gas}^{(n+1)}-\hat{E}_{\rm gas}^{(n)}}{\Delta \hat{t}} \\
&&\quad -\,\frac{\mathcal {C}}{2} \left(\hat{\nabla }\cdot \hat{\boldsymbol{F}}^{(n)} + \hat{\nabla }\cdot \hat{\boldsymbol{F}}^{(n+1/2)} \right).
\end{eqnarray*}

Substituting equation (67) and equation (68) into equation (69), we obtain


(70) \begin{eqnarray*}
&& \frac{\hat{E}^{(n+1)}-\hat{E}^{(n)}}{\Delta \hat{t}} + \mathcal {P}^{-1}\frac{\hat{E}_{\rm gas}^{(n+1)}-\hat{E}_{\rm gas}^{(n)}}{\Delta \hat{t}} \\
&&\quad = \frac{1}{2} \left[\left(\frac{1}{2}\right) \frac{\mathcal {C}}{3\mathcal {L}} (\hat{\nabla }^2\hat{E}^{(n)} + \hat{\nabla }^2 \hat{E}^{(n-1/2)}) + \frac{\mathcal {C}^2 \Delta \hat{t}}{3} \hat{\nabla }^2 \hat{E}^{(n)} \right].
\end{eqnarray*}

Thus we see that there are two modes of radiation diffusion in this numerical scheme: one is physical diffusion with a diffusion coefficient of $\mathcal {C}/3\mathcal {L}$, and the other is numerical diffusion with a coefficient of $\mathcal {C}^2 \Delta \hat{t}/3$. This numerical mode will dominate for any time-step $\Delta \hat{t}\gtrsim 1/\mathcal {C}\mathcal {L}$, or, in dimensional terms, Δ*t* > λ_∞_/*c*, i.e. whenever the time-step is large enough that we do not resolve the light-crossing time of a photon mean-free path. In the asymptotic diffusion regime, where the photon mean-free path is smaller than the size of a cell, this means that numerical diffusion dominates any time that the time-step is larger than the light crossing time of a cell. In practice, the time-step is always much larger than this – since otherwise one might as well use a fully explicit scheme – which explains why discretizations such as RK2-SSP fail in the asymptotic diffusion regime.

Comparing equation (70) to equation (62), we see that the numerical diffusion mode is removed in the IMEX discretization, and by comparing the calculations leading up to these equations we can also understand why. The numerical diffusion term in the RK2-SSP update originates in a term that appears at the intermediate stage of the flux update (equation 67). This term does not appear to leading order in the intermediate stage of the IMEX update (equation 57) because it is overwhelmed by the source term, which is a factor of $\mathcal {L}$ larger; it is a cancellation within the source term that forces the radiation flux to the correct value for diffusion. Thus the IMEX update winds up with an estimate for the intermediate-state flux that is of order $1/\mathcal {L}$ independent of the time-step, while the RK2-SSP update, because it ignores the order $\mathcal {C}\mathcal {L}$ source term but retains the order $\mathcal {C}$ transport term during the intermediate stage, obtains a flux estimate that is of order $\mathcal {C}\Delta \hat{t}$ instead. This overestimate is what gives rise to the artificial numerical diffusion of energy.

An important conclusion to draw from this argument is that the failing in the RK2-SSP scheme for this problem is not specific to that scheme, but is instead generic to *any* update scheme that contains a stage that includes only the transport terms and not the source terms, or where the source terms are operator-split from the transport terms (e.g. Jiang et al. 2014; Skinner et al. 2019). Such a scheme will always overestimate the transport in diffusion-regime problems where the source term is responsible for suppressing them.

### 4.3 On modifications to the radiation wave speed

The problem we have identified in the RK2-SSP scheme is not new; indeed, multiple authors have pointed out that the HLL solver applied to the radiation subsystem, in a scheme where the source and transport terms are operator-split, yields fluxes that fail to preserve the asymptotic diffusion limit (Lowrie & Morel 2001; Audit et al. 2002; Jiang et al. 2013; Skinner et al. 2019; Wibking & Krumholz 2022). In an attempt to circumvent this problem, these authors have proposed a range of corrections to the energy fluxes; for instance, Skinner et al. (2019) suggest


(71) \begin{eqnarray*}
\mathcal {F}_{E, \text{ corrected }}^{\mathrm{HLL}}=\frac{S_\mathrm{ R} F_\mathrm{ L}-S_\mathrm{ L} F_\mathrm{ R}+\epsilon S_\mathrm{ R} S_\mathrm{ L}\left(E_\mathrm{ R}-E_\mathrm{ L}\right)}{S_\mathrm{ R}-S_\mathrm{ L}},
\end{eqnarray*}

where *S*_L,R_ are the characteristic left and right wavespeeds, respectively, whose values absent the RSLA are given by $S = \pm \sqrt{f} c$, where *f* is the component of the Eddington tensor along that direction. In this expression, ϵ is an empirical correction factor that smoothly transitions from 1 in the streaming limit to 1/τ in the optically thick limit, where τ is an estimate of the optical depth, usually computed as the optical depth across *N* computational cells. The value of *N* must be determined empirically by testing the code, and is not the same for all codes or numerical schemes: for example Skinner et al. (2019) use *N* = 1, while Jiang et al. (2013) use $N=\sqrt{10}$. This correction implies an effective wave speed of


(72) \begin{eqnarray*}
S_{\rm eff} = c \sqrt{\frac{f}{\tau }},
\end{eqnarray*}

In light of the preceding discussion, we can see that this reduction in the flux is effectively a correction that attempts to reduce the numerical diffusion mode in equation (70), by in turn forcing the overestimated intermediate time–flux (equation 67) back toward the solution that would have been obtained by retaining rather than dropping the source term. However, the accuracy of this fix, particularly in the regime of intermediate optical depth, is not known.

In our IMEX PD-ARS method, we use the wavespeed *S* without any correction in the Riemann solver. No correction is necessary because, by retaining the source term, we automatically recover the correct flux in the diffusion limit (equation 57), and our scheme correctly and smoothly goes between the optically thin limit, where the transport term dominates, and the optically thick limit, where the source term does. Indeed, a corollary of this analysis is that, in the highly optically thick regime where the source term is dominant, we need not even obey the Courant–Friedrichs–Lewy (CFL) condition for the radiation in order to obtain the correct answer. To understand why this is the case, we notice that equation (62) is an effective temporal discretization of the diffusion equation


(73) \begin{eqnarray*}
\frac{\partial }{\partial \hat{t}} \hat{E}= \frac{\mathcal {C}}{3 \mathcal {L}} \hat{\nabla }^2 \hat{T}^4,
\end{eqnarray*}

which has a diffusion coefficient of $\hat{D}_{\rm diff} = \mathcal {C}/(3\mathcal {L})$. The effective speed of the diffusion in time interval $\hat{t}$ is approximately


(74) \begin{eqnarray*}
\hat{S}_{\rm diff} \approx \frac{\sqrt{\hat{D}_{\rm diff} \hat{t}}}{\hat{t}} = \sqrt{\frac{\mathcal {C}}{3 \mathcal {L}\hat{t}}}.
\end{eqnarray*}

To better understand the magnitude of $\hat{S}_{\rm diff}$, we reintroduce dimensions to the variables and get


(75) \begin{eqnarray*}
S_{\rm diff} = \sqrt{\frac{c}{3 t \chi _0}} = \frac{c}{\sqrt{3}} \frac{1}{\sqrt{{\rm CFL} ~\tau _{\rm cell}}},
\end{eqnarray*}

where CFL ≡ *ct*/Δ*x* is the radiation CFL number. This evaluation of diffusion speed aligns with the wave speed correction ‘hack’ (equation 72) when CFL  = 1. Our IMEX scheme’s asymptotic preserving nature, as demonstrated, allows the CFL number to exceed 1 while still accurately capturing the diffusion limit. This is exemplified in the subsequent discussion on the asymptotic Marshak wave test (Section 5.3), where our numeric scheme accurately catches the position of the diffusion front for radiation CFL numbers up to ∼10. This observation is in concordance with Jiang et al. (2013), who, through a linear analysis of the diffusion equations, showed that if one calculates the effective propagation speed with a wavelength of 10 cells, the numerical diffusion is small enough not to affect the solution. This compatibility with the findings from linear wave analysis of diffusion equations not only validates our scheme’s robustness in limiting numerical diffusion effectively but also underscores the versatility of the IMEX PD-ARS scheme in accommodating higher CFL numbers without compromising the asymptotic preserving property.

## 5 NUMERICAL TESTS

The original quokka paper ([Bibr bib37][Bibr bib37]) introduced a series of tests to validate the accuracy and convergence capabilities of quokka, including tests of the hydrodynamic subsystem, the radiation transport, and coupled RHD. This new scheme passes all these tests, so we will not repeat them here, with the exception of one test that we repeat to demonstrate the convergence properties of our scheme. We instead introduce additional tests for our implementation of the IMEX PD-ARS scheme in quokka that are specifically designed to test the code’s ability to preserve the asymptotic diffusion limit. All these tests use the Levermore (1984) closure for the RHD system – see [Bibr bib37][Bibr bib37] for full details of the implementation. The full source code and outputs for all tests are available in the quokka github repository (see Data Availability statement for details).

### 5.1 Convergence rate test

We demonstrate the convergence rate of our RHD solver through the optically thin radiation-driven wind test introduced by [Bibr bib37][Bibr bib37]; in this test a radiation field passes through and accelerates an optically thin gas. We setup this test as described in Section 3.3.2 of [Bibr bib37][Bibr bib37], with the exception that the spatial resolution varies from *N_x_* = 1024 to 8192 grid cells and the CFL number varies from 0.1 to 0.6; we use much higher spatial resolution than in the original test in order to reach a limit where the temporal rather than the spatial discretization dominates the error, and we choose an optically thin problem because we only expect second-order temporal convergence in the streaming limit.

**Figure 1.**
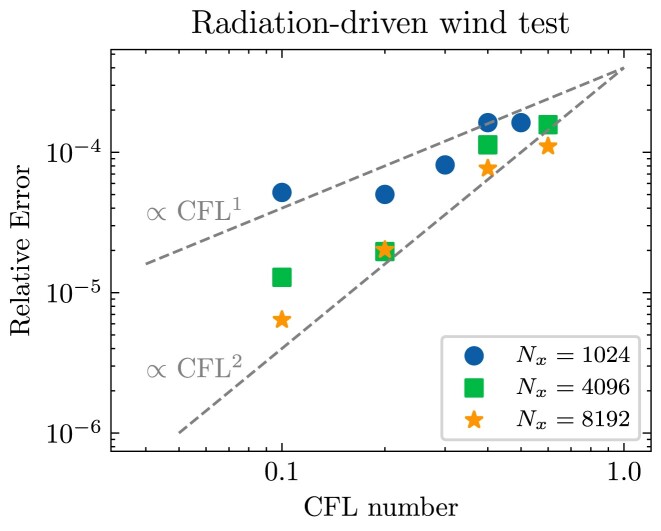
Relative L1 error as a function of CFL number for three different spatial resolutions *N_x_* = 1024, 4096, and 8192, as indicated in the legend, for the optically thin radiation-driven wind test. Dashed lines show slopes of 1 and 2 to guide the eye. For sufficiently large spatial resolution the convergence rate approaches quadratic as expected from the second-order scaling of our time-integration scheme in the streaming limit.

We show the error of our numerical solution relative to the exact solution (again computed exactly as in [Bibr bib37][Bibr bib37]) in Fig. 1. At fixed *N_x_*, the relative error decreases with decreasing CFL number, or equivalently, time-step size. As the accuracy of the numerical solution depends on both spatial and temporal resolutions, we expect that the convergence rate with respect to the CFL number to steepen as spatial resolution increases. This is confirmed in the figure. At *N_x_* = 8192, the power-law slope of the error-CFL number scaling relation converges towards the value of 2 expected for the second-order accuracy of our time-integration scheme in the streaming limit. This confirms that our implementation achieves the theoretically expected convergence rate.

### 5.2 Non-equilibrium radiation shock

**Figure 2.**
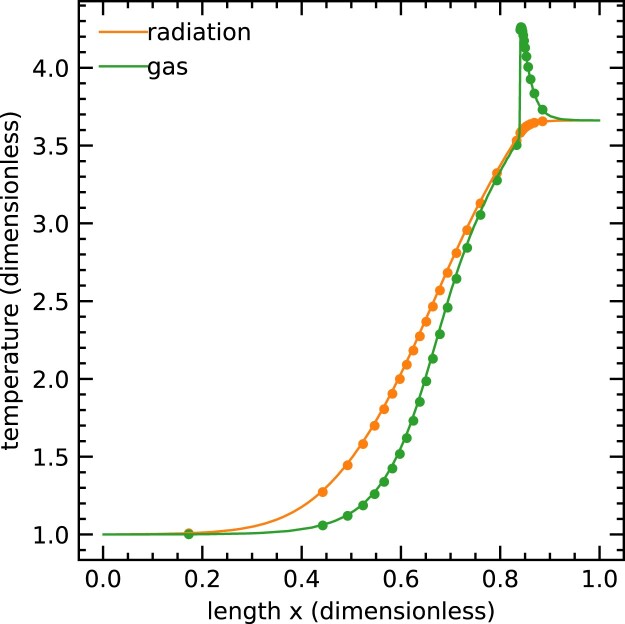
Comparing radiation and gas temperature from the numerical calculation (solid lines) with the exact steady-state solution (dots) in a subscritical radiation shock with *M* = 3.

We begin our tests in the diffusion regime with the classical grey non-equilibrium radiative shock test described by Zel’dovich & Raizer (2012). Radiation can modify the structure of a shock because it diffuses and interacts with matter. Lowrie & Edwards (2008) have found a semi-analytic exact solution of radiative shocks with grey non-equilibrium diffusion. Using their parameters with an upstream Mach number of ${\cal M} = 3$, we obtain a subcritical radiation shock whose temperature jumps discontinuously at the shock interface. We present our numerical calculation of this problem and compare them to the solutions of Lowrie & Edwards (2008). Following the setup used by Skinner et al. (2019), we scale from dimensionless to cgs units by setting the opacities to κ = 577 cm^2^ g^−1^ and mean molecular weight to μ = *m*_H_. We use an adiabatic equation of state with an adiabatic index to γ = 5/3. The shock is simulated in a 1D region with *x* ∈ [0, 0.015 75] cm resolved with 512 grids. The problem is set in the rest frame of the shock initialized at *x* = 0, and the conditions on the left and right sides of the shock are uniform, with densities, temperatures, and velocities given by ρ_L_ = 5.69 g cm^−3^, *T*_L_ = 2.18 × 10^6^ K, *v*_L_ = 5.19 × 10^7^ cm s^−1^, and ρ_R_ = 17.1 g cm^−3^, *T*_R_ = 7.98 × 10^6^ K, *v*_R_ = 1.73 × 10^7^ cm s^−1^, respectively. In order to exactly match the assumptions used in the semi-analytic solution, we use the Eddington approximation, $\mathsf {\mathit{ P}}= (1/3) E \mathsf {I}$, to calculate the radiation pressure tensor. Following Skinner et al. (2019), we use a reduced speed of light $\hat{c} = 10 (v_\mathrm{ L} + c_{\mathrm{ s},\mathrm{ L}})$, where *c*_s,L_ is the adiabatic sound speed of the left-side state. We use a CFL number of 0.4 and evolve until *t* = 10^−9^ s. We show the resulting temperature profile in Fig. 2. The agreement between our numerical calculation and the semi-analytic solution is excellent. The *L*_1_ norm of the relative error of the gas temperature is 0.38 per cent, which is as good as the solution of Skinner et al. (2019).

### 5.3 The asymptotic Marshak-wave problem

We test the code’s ability to accurately capture radiation in the optically thick limit via the diffusive Marshak-wave problem. This problem consists of a semi-infinite medium of material with a variable absorption coefficient χ = 300 cm^−1^/(*k*_B_*T*/keV)^3^. We input incoming isotropic radiation on the boundary at *x* = 0 from a 1 keV temperature source. We use an initial condition of *T*(*x*) = *T*_0_ = 10^−3^ keV and $E(x) = a_\mathrm{ r} T_0^4$ for our test problem.

**Figure 3.**
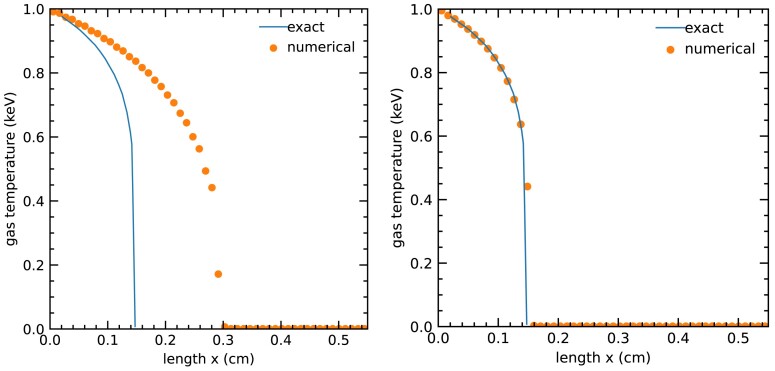
Comparing numerical solutions to the Marshak-wave problem in the asymptotic diffusion limit from the SSP-RK2 scheme (left) and IMEX PD-ARS scheme we introduce here (right). The solid lines are the semi-analytic solutions and the dots are the nodal values of the numerical solution. Both simulations run with Δ*x* = 0.011 cm and CFL =0.9. The IMEX scheme accurately captures the diffusion of radiation while in the SSP-RK2 scheme solution, the wavefront propagates about two times too fast.

Fig. 3 shows our numerical results compared to the semi-analytic solution for the diffusive Marshak-wave problem, alongside a comparison to the solution using the SSP-RK2 scheme. The numerical calculation uses a spatial grid of 60 cells across the domain (0, 0.66 cm), with each cell spanning 3 × 10^9^ mean-free paths at the minimum temperature and three mean-free paths at the highest temperature. The heat capacity in this problem is constant at 3 × 10^15^ erg cm^−3^ keV^−1^. We compare this solution with the semi-analytic equilibrium-diffusion solution from Zel’dovich & Raizer (2002). We find a relative L1-norm error at about $4.5~{{\ \rm per\ cent}}$. The simulation results presented here are obtained using a CFL number of 0.9, but the simulation runs with a CFL number up to ∼10, implying a time–step roughly 10 times the largest permissible time-step in the streaming limit.

### 5.4 Advecting radiation pulse in the static diffusion limit

In this test, introduced by Krumholz et al. (2007), we simulate the advection of a radiation pulse by the gas motion in the diffusion regime. We set the initial gas and radiation temperatures equal in this test, and the initial temperature and density profiles are


\begin{eqnarray*}
&& T=T_0+\left(T_1-T_0\right) \exp \left(-\frac{x^2}{2 w^2}\right), \\
&& \rho =\rho _0 \frac{T_0}{T}+\frac{a_R \mu }{3 k_{\mathrm{B}}}\left(\frac{T_0^4}{T}-T^3\right),
\end{eqnarray*}

with *T*_0_ = 10^7^ K, *T*_1_ = 2 × 10^7^ K, ρ_0_ = 1.2 g cm^−3^, *w* = 24 cm, and μ = 2.33*m*_p_ = 3.9 × 10^−24^ g. The radiation pressure is estimated via the Eddington approximation. The opacity of the gas is set at κ_0*P*_ = κ_0*E*_ = κ_0*F*_ = 100 cm^2^ g^−1^. The system is initially in pressure balance. If there were no radiation diffusion, the system would be in an equilibrium between the gas pressure and radiation pressure. Because of radiation diffusion, the balance is lost and the gas moves.

**Figure 4.**
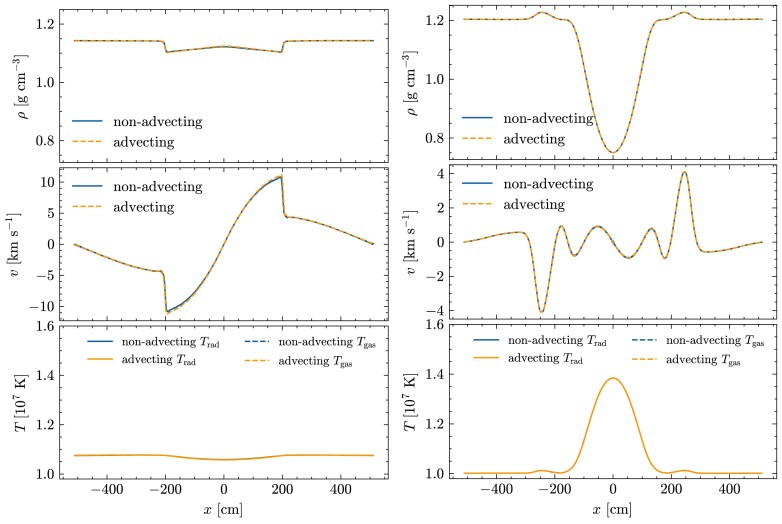
Advecting radiation pulse tests in the static diffusion regime at *t* = 4.8 × 10^−5^ s using the RK2-SSP scheme (left) versus our newly introduced IMEX scheme (right). The IMEX scheme accurately captures the radiation diffusion, agreeing well with the results presented in Krumholz et al. (2007) and Zhang et al. (2011).

**Figure 5.**
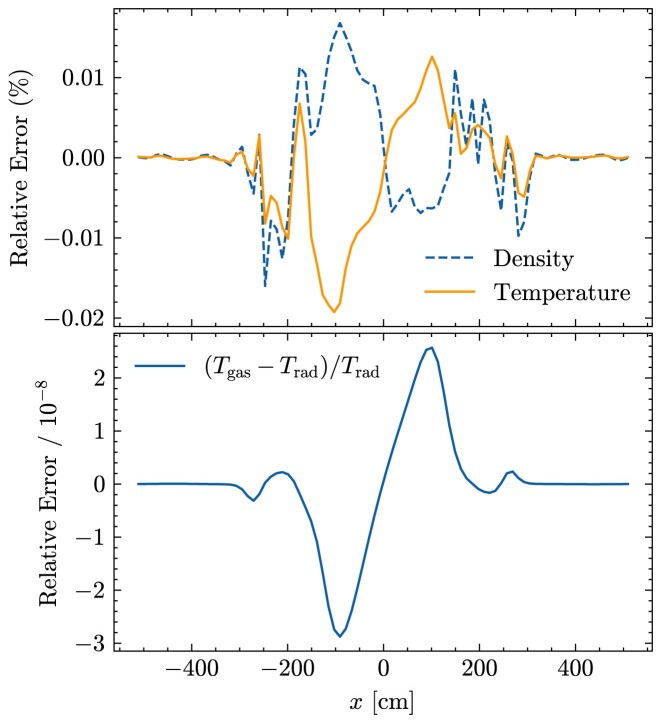
Top: relative errors in density (dashed lines) and temperature (solid lines) between the advected and unadvected runs. Bottom: relative difference between gas and radiation temperature in the advected run.

We solve the problem numerically in two different frames, one in the lab frame and the other in the comoving frame, and compare the results in Fig. 4. In the comoving frame run, the velocity is 0 in the beginning everywhere. In the lab frame run, the initial velocity is *v*_0_ = 10^6^ cm s^−1^. In both runs, the optical depth across the pulse is τ = ρκ*w* = 2.9 × 10^3^, and in the lab-frame run β ≡ *v*_0_/*c* = 3.3 × 10^−5^, giving βτ ≈ 0.1 and placing this problem in the static diffusion limit. The computational domain in both runs is a 1D region of (−512, 512 cm) with periodic boundaries, and the grid consists of 512 uniform cells. We show the density, temperature, and velocity profiles from both runs at *t* = 4.8 × 10^−5^ s in Fig. 4. The results of the lab-frame run have been shifted in space by *v*_0_*t* for comparison. The density, temperature, and velocity profiles from both runs are almost identical, demonstrating the accuracy of our scheme in capturing radiation advection. We also show the relative difference from both runs in Fig. 5. The relative difference between the non-advecting and advecting cases is below $0.03~{{\ \rm per\ cent}}$ anywhere. The relative difference between the temperature of the gas and radiation is below 3 × 10^−9^. We compare these results with that of Krumholz et al. (2007) and Zhang et al. (2011) and find good agreement.

### 5.5 Advecting radiation pulse in the dynamic diffusion limit

**Figure 6.**
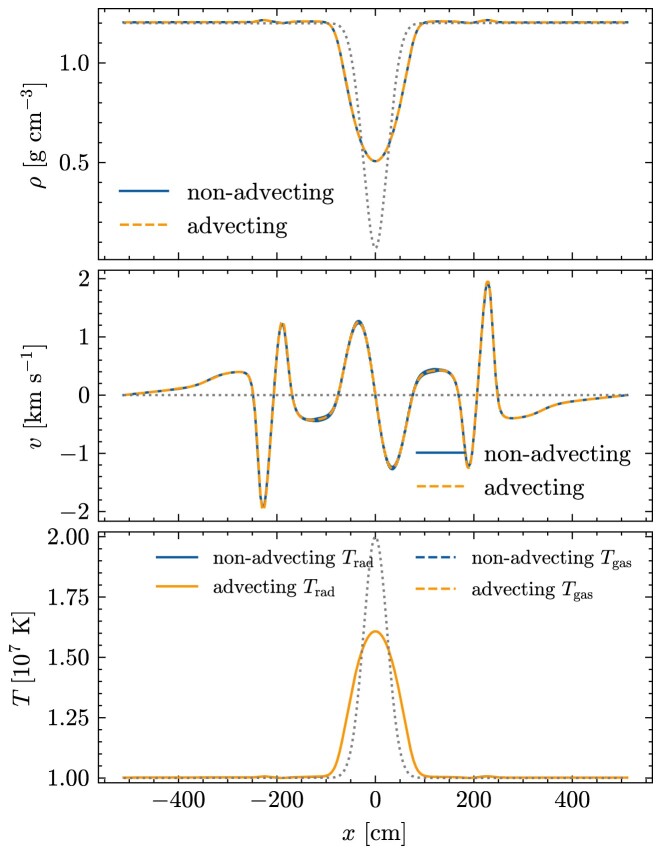
Advecting radiation pulse test in the dynamic diffusion regime, where β ≡ *v*/*c* = 10^−3^ and the optical depth across the pulse is τ = 10^4^, yielding βτ = 10. The initial condition is plotted as grey dotted lines. The observed radiation diffusion closely matches the theoretical predictions derived from the diffusion equation. The difference between the advected and non-advected pulse is so small that it is nearly invisible to the eye.

In this test, we rerun the advecting radiation pulse problem in the dynamic diffusion regime. Compared to the static diffusion case, the advection speed is increased to 3 × 10^7^ cm s^−1^ and the opacity is increased to 500 cm^2^ g^−1^. Numerically, β = 10^−3^, τ = 1.4 × 10^4^, and βτ = 14, placing this problem in the dynamic diffusion limit. We increase the number of cells to 1024 to reduce the magnitude of odd–even decoupling instability, and evolve the simulation to *t* = 4.8 × 10^−5^ s. The relative difference between the non-advecting and advecting cases is below 0.06 per cent anywhere (Fig. 6), demonstrating the excellent accuracy of our scheme in capturing radiation advection in the dynamic diffusion regime. Although there is no analytic solution to this problem, we can estimate the expected pulse width via the diffusion equation


(76) \begin{eqnarray*}
\frac{\partial }{\partial t} E = \frac{c}{3\chi } \frac{\partial ^2}{\partial x^2} E,
\end{eqnarray*}

The expected distance that the pulse has diffused at time *t* is approximately


(77) \begin{eqnarray*}
w \approx \sqrt{\frac{c t}{3\chi }} = 28.3 ~{\rm cm},
\end{eqnarray*}

The temperature profile of our numerical calculation Fig. 6 agrees well with this analytic estimation.

### 5.6 Advecting uniform medium in the dynamic diffusion regime

We test the code’s ability to incorporate relativistic corrections up to order *v*/*c* or order *v*^2^/*c*^2^ with a 1D test problem of advecting a uniform medium in the dynamic diffusion regime. In this test, the gas is initially moving at a uniform velocity of *v*_0_ = 0.01*c* and has a uniform density. We configure the opacity and grid size such that each grid’s optical depth is 10^5^. The radiation is initially in thermal equilibrium with the medium at the comoving frame.

In the lab frame, to order *v*/*c* the initial radiation quantities are


(78) \begin{eqnarray*}
E(t=0) = E_0,
\end{eqnarray*}

(79) \begin{eqnarray*}
F(t=0) = \frac{4}{3} v_0 E_0,
\end{eqnarray*}

and we can show that the space-like component of the radiation four-force, equation (4), to order *v*/*c* vanishes:


(80) \begin{eqnarray*}
-G_1 =-\chi _{0} \frac{{F}}{c} + \chi _{0} \frac{4 \pi B}{c} \frac{v}{c} + \chi _{0} \frac{{v_j} P_{j1}}{c}
\end{eqnarray*}

(81) \begin{eqnarray*}
\quad = -\chi _{0} \frac{4}{3} E_0 \frac{v_0}{c} + \chi _{0} E_0 \frac{v_0}{c} + \chi _{0} \frac{1}{3} E_0 \frac{v}{c}
\end{eqnarray*}

(82) \begin{eqnarray*}
\quad = 0
\end{eqnarray*}

To order *v*^2^/*c*^2^ the initial radiation quantities are


(83) \begin{eqnarray*}
E(t=0) = E_0 \left(1 + \frac{4}{3} \frac{v^2}{c^2}\right),
\end{eqnarray*}

(84) \begin{eqnarray*}
F(t=0) = \frac{4}{3} v_0 E_0,
\end{eqnarray*}

and we can show that both the time-like and space-like component of the radiation four-force vanishes:


(85) \begin{eqnarray*}
-c G_0 & =&c \chi _0 \left(\frac{4 \pi B}{c}-E\right)\left(1+\frac{1}{2}\frac{v^2}{c^2}\right) + \chi _{0} \frac{v_i F_i}{c} \\
& =& c \chi _0 \left[E_0-E_0 (1 + \frac{4}{3} \frac{v^2}{c^2}) \right] \left(1+\frac{1}{2}\frac{v^2}{c^2}\right) + \chi _{0} \frac{v_0}{c} \frac{4}{3} v_0 E_0 \\
& =& O\left(\frac{v^4}{c^4}\right)
\end{eqnarray*}

(86) \begin{eqnarray*}
-G_1 & =&-\chi _{0F} \frac{{F}}{c} \left(1+\frac{1}{2} \frac{v^2}{c^2}\right) + \chi _{0P} \frac{4 \pi B}{c} \frac{v}{c} + \chi _{0F} \frac{{v_j} P_{j1}}{c} \\
& =& -\chi _{0F} \frac{4}{3} E_0 \frac{{v_0}}{c} \left(1+\frac{1}{2} \frac{v_0^2}{c^2} \right) + \chi _{0P} E_0 \frac{v_0}{c} \\
&& +\,\chi _{0F} \frac{{v_0}}{c} \frac{1}{3} E_0 (1 + \frac{4}{3} \frac{v_0^2}{c^2}) \\
& =& O\left(\frac{v^3}{c^3}\right)
\end{eqnarray*}

**Figure 7.**
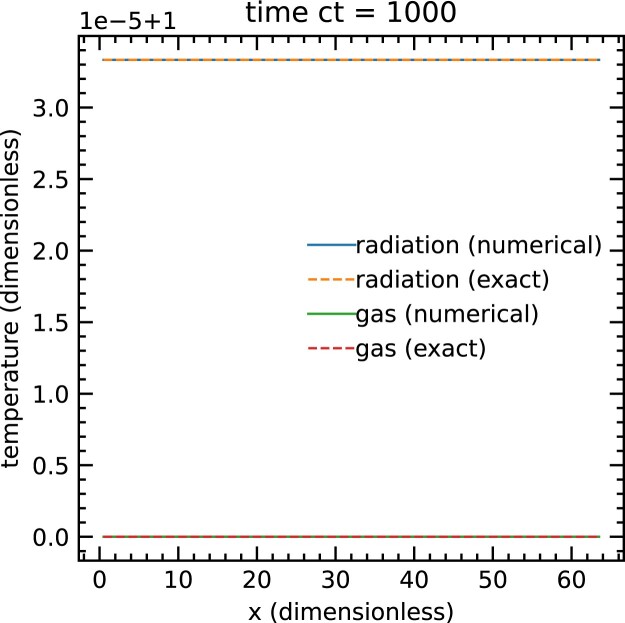
Advecting uniform medium test in the dynamic diffusion limit (β = 0.01, βτ = 10^3^). The numerical results match the analytic solution to the limits of machine precision.

The key feature of this test is that, in the lab frame, the radiation energy is out of equilibrium with the thermal radiation. However, this excess is offset by the work done to the radiation by the matter. One can show that our backwards Euler scheme, equation (42) and equation (43), preserves this equilibrium, leading to an equilibrium state (*E*^(*n* + 1)^ = *E*^(*n*)^ and $\boldsymbol{F}^{(n+1)} = \boldsymbol{F}^{(n)}$) that aligns precisely with the expected outcomes of this test (Fig. 7), achieving precision up to machine accuracy even in the dynamic diffusion limit (Table 1). Conversely, the source term from Howell & Greenough (2003) and [Bibr bib37][Bibr bib37], defined as ∝ (4π*B*/*c* − *E*_r_), fails to attain this balance.

**Table 1.** Relative errors of the matter temperature in numerical calculation with respect to theoretical solution in the advecting uniform medium tests. Col. 3: the *v*/*c* order used in the source terms.

β	βτ	Order	Error
10^−4^	1	1	<10^−15^
10^−2^	10^3^	1	4 × 10^−5^
10^−2^	10^3^	2	<10^−15^

## 6 CONCLUSION

We have presented a novel mixed IMEX time integration scheme for finite-volume RHD that is second-order accurate in the streaming limit and accurately preserves the asymptotic diffusion limit. Our method uniquely combines the robustness of *local* implicit methods in handling stiff source terms with the scalability and simplicity of explicit methods for advective transport, making it particularly suitable for adaptive mesh refinement and massively parallel, GPU-accelerated architectures. This scheme addresses a critical gap in current RHD solvers by ensuring asymptotic accuracy in the streaming, static diffusion, and dynamic diffusion limits, including in the asymptotic diffusion regime where the photon mean-free path is much smaller than the computational grid, without necessitating non-local implicit steps or ad hoc adjustments to the radiation flux based on the optical depth. We have implemented our algorithm in the GPU-accelerated AMR RHD code quokka ([Bibr bib37][Bibr bib37]). quokka is open-source and a link to download the source code is in the DATA AVAILABILITY section.

We have verified our algorithm using a variety of established quantitative tests, including the non-equilibrium radiation shock test, the asymptotic Marshak wave test, and the advecting radiation pulse test in the static and dynamic diffusion limit. These tests demonstrate the scheme’s capability to recover the correct asymptotic limits.

Looking forward, we envision several areas for further development. First, extending our scheme to include more complex radiation transport models, such as multifrequency radiation transfer and non-thermal emission, could expand its applicability to a broader range of astrophysical problems. In addition, integrating our RHD method with other physics modules, such as magnetic fields, could also open new channels for multiphysics simulations in astrophysics. Finally, although convex-invariant IMEX methods that achieve second- or higher-order accuracy in the diffusion regime but have time-steps restricted solely by the transport terms do not exist (see Gottlieb, Shu & Tadmor 2001; Chu et al. 2019), it is possible to generalize our scheme to achieve higher order accuracy in the streaming limit by including additional RK stages. We will also investigate this possibility in future work.

## Data Availability

The quokka code, including the source code and summary result plots for all tests presented in this paper, is available from https://github.com/quokka-astro/quokka under an open-source license.

## Footnotes

1 While in this paper we focus on RHD for simplicity, our method applies equally well to radiation-magnetohydroynamic systems and equations.

2 Note that we do not set the reference length scale for radiation quantities to ℓ because we wish to ensure that $\hat{\chi }_0$ is a quantity of order unity, and ℓ and λ_∞_ may need to be very different in order to accomplish this.

3 Of course we have shown above that, due to cancellations, these terms in fact wind up being of the same order – but our goal is precisely for our numerical method to be able to recover this cancellation.

4 By convex-invariant, we mean that the scheme has the property that if the forwards Euler update produces states that are bounded by a convex set (e.g. the update guarantees that the internal energy remains positive, or that the total variation is diminishing) then the full multistage update also remains within that bound. For a discussion of why this property is advantageous, see Gottlieb & Shu (1998).

5 Formally, Chu et al. (2019) show that IMEX PD-ARS reduces to RK2-SSP when $\boldsymbol{S}$ is small compared to $\boldsymbol{T}$, which is the case in the streaming limit. By contrast the scheme has only first order accuracy in the diffusion limit.

6 Note that the total energy in dimensionless units is $\hat{E}+ \mathcal {P}^{-1} \hat{E}_\mathrm{gas}$ rather than $\hat{E}+ \hat{E}_\mathrm{gas}$ because in our non-dimensionalization the matter and radiation energies are scaled differently – the matter energy is normalized by $\rho _\infty a_\infty ^2$, while the radiation energy is normalized by $a_r T_\infty ^4$, and these two differ by a factor of $\mathcal {P}$. See Section 2.2 for details.

7 Note that in writing down these equations we assume that our closure for the pressure tensor will produce $\mathsf {\mathit{ P}}\rightarrow (1/3)\mathsf {I}E$ in optically thick conditions, which is true of any reasonable closure scheme.
